# Prognostic implications of N^6^-methyladenosine RNA regulators in breast cancer

**DOI:** 10.1038/s41598-022-05125-x

**Published:** 2022-01-24

**Authors:** Jiaojiao Tai, Linbang Wang, Hao Guo, Ziqiang Yan, Jingkun Liu

**Affiliations:** 1 Department of Orthopedics, Honghui Hospital, Xi’an Jiaotong University, No. 555, Youyi Road, Beilin District, Xi’an, 710054 Shaanxi People’s Republic of China; 2 Department of Orthopedic Surgery, The First Affiliated Hospital, Chongqing Medical University, Chongqing, 400016 People’s Republic of China

**Keywords:** Breast cancer, Tumour immunology

## Abstract

The significance of N^6^-methyladenosine (m6A) RNA modifications in the progression of breast cancer (BC) has been recognised. However, their potential role and mechanism of action in the tumour microenvironment (TME) and immune response has not been demonstrated. Thus, the role of m6A regulators and their downstream target gene components in BC remain to be explored. In this study, we used a series of bioinformatics methods and experiments to conduct exploratory research on the possible role of m6A regulators in BC. First, two regulatory modes of immune activation and inactivation were determined by tumour classification. The TME, immune cell infiltration, and gene set variation analysis results confirmed the reliability of this pattern. The prognostic model of the m6A regulator was established by the least absolute shrinkage and selection operator and univariate and multivariate Cox analyses, with the two regulators most closely related to survival verified by real-time quantitative reverse transcription polymerase chain reaction. Next, the prognostic m6A regulator identified in the model was crossed with the differential copy number of variant genes in invasive BC (IBC), and it was determined that YTHDF1 was a hub regulator. Subsequently, single-cell analysis revealed the expression patterns of m6A regulators in different IBC cell populations and found that YTHDF1 had significantly higher expression in immune-related IBC cells. Therefore, we selected the intersection of the BC differential expression gene set and the differential expression gene set of a cell line with knocked-down YTHDF1 in literature to identify downstream target genes of YTHDF1, in which we found IFI6, EIR, and SPTBN1. A polymerase chain reaction was conducted to verify the results. Finally, we confirmed the role of YTHDF1 as a potential prognostic biomarker through pan-cancer analysis. Furthermore, our findings revealed that YTHDF1 can serve as a new molecular marker for BC immunotherapy.

## Introduction

Breast cancer (BC), a cancer of epithelial origin, accounts for 25% of all diagnosed cancer cases and 15% of cancer deaths in women^1^. Traditional risk factors for BC include clinical features of the tumour, such as stage and pathological classification, and the patient’s age at diagnosis. While these factors have been widely used in the evaluation of prognosis and treatment^2^, new predictive indicators at the molecular level are still lacking^3^. Therefore, it is crucial to discover the molecular mechanism of BC progression in order to identify prognostic biomarkers and develop effective therapies^4^.

Genetic and epigenetic regulation in the tumour cell have been shown to participate in almost every aspect of tumourigenesis and cancer progression^5^. RNA modifications are known to have the most abandon types of varieties of more than 150, in which N^6^-methyladenosine (m6A) is recognised as an important universal modifier in eukaryotes^6^. Regulators of m6A are a group of proteins that alter the performance of m6A^7^ and have been categorised as ‘writers’, ‘erasers’, and ‘readers’, according to their functions^8^. Moreover, m6A modification has been reported to be involved in embryonic development and various types of cancer^7^.

Immunological checkpoint blockade-based immunotherapy has exhibited remarkable efficacy in clinical applications^9^. However, success varies with individuals, making it difficult to address clinical needs^10^. The tumour microenvironment (TME) is considered to play a crucial role in tumour progression^11^, and contains not only cancer cells but also macrophages, and other recruited immune cells, including myeloid cells and lymphocytes^12^. Biological changes, such as angiogenesis, hypoxia avoidance, and immune tolerance induction, can be elicited by cancer cells depending on the TME components^13,14^. Characterisation of the TME cell infiltrate is a key component for predicting patient immunotherapeutic responsiveness and for identifying novel therapeutic targets^15^. Zhang et al. showed a correlation between TME immune cells and m6A modifications^16^. Han et al. reported that YTHDF1 enhanced tumour infiltrating CD8 + T cell antitumour responses by affecting the transcripts encoding lysosomal proteases that are modified by m6A methylation. Additionally, YTHDF1 inhibition improved the therapeutic efficacy of anti-PDL1 blockade^17^.

**Figure 1:**
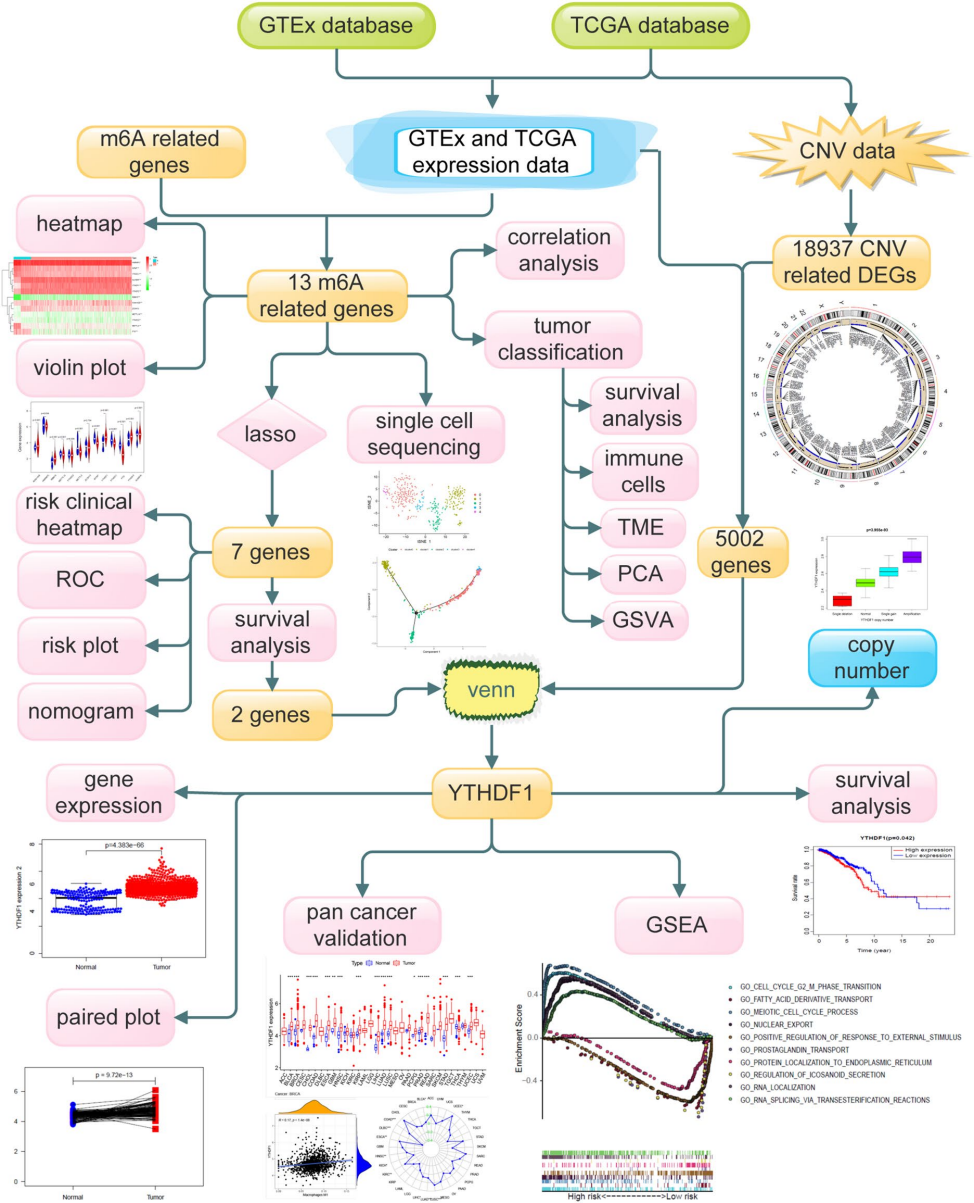
A flowchart showing the steps in this study.

In this study, we first typed BC samples based on m6A regulators, and observed the correlation of different types with TME and immune cell infiltration. Subsequently, univariate Cox and least absolute shrinkage and selection operator (LASSO) analysis was used to construct a prognostic model of m6A regulators. Next, single-cell analysis revealed the expression patterns of m6A regulators in different invasive BC (IBC) cell populations. Finally, the prognostic m6A regulator in the model was hybridised with the differential copy number variants (CNVs) genes in IBC, with YTHDF1 being a pivotal regulator. Furthermore, the expression and function of YTHDF1 were verified using a variety of methods. A schematic diagram showing the steps in this study is shown in Fig. 1, and the diagram was drawn using Edraw (versions 9.4).

## Results

### Tumour classification

**Figure 2:**
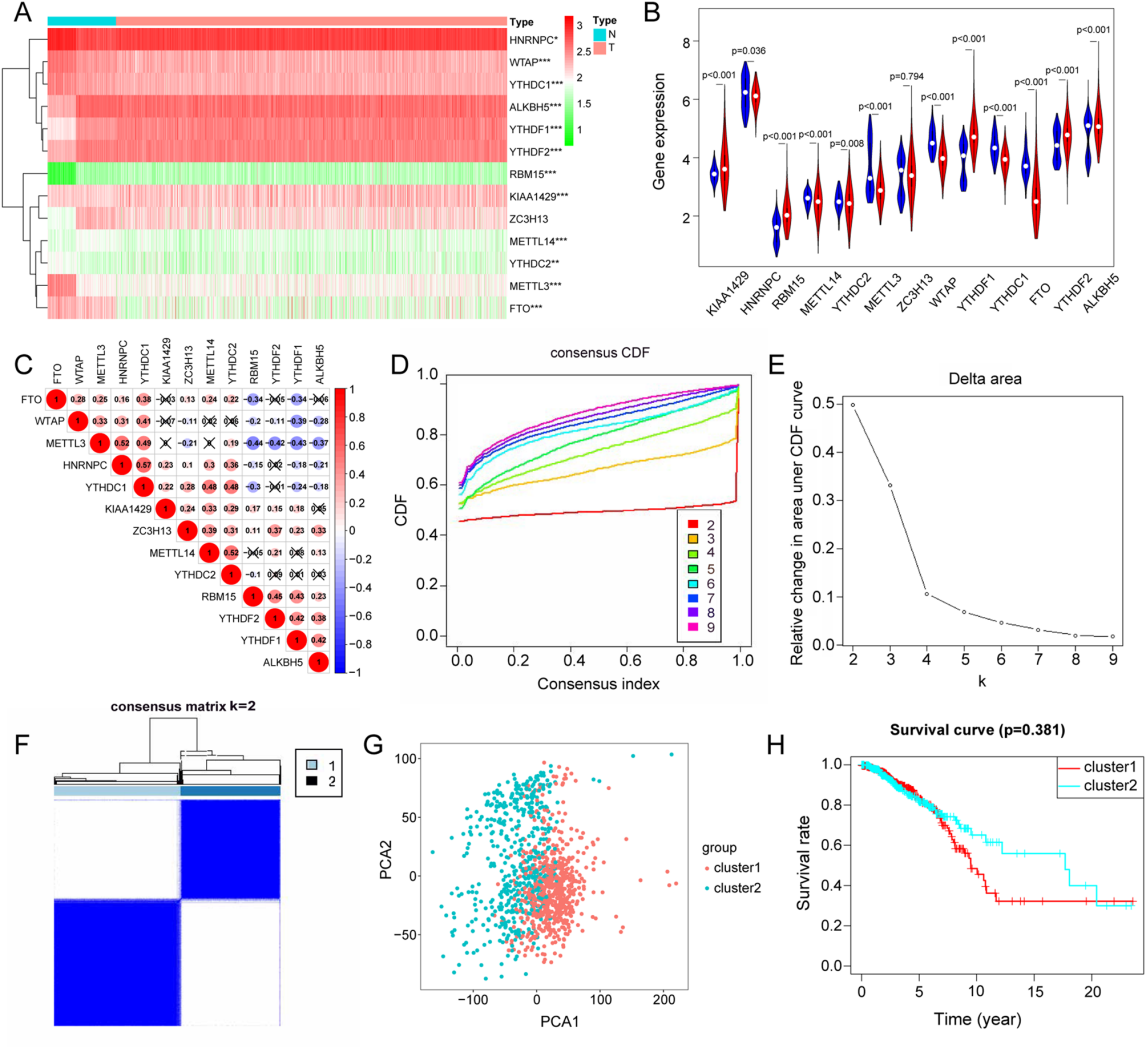
The expression of m6A regulator genes and clustering of samples. (**A**) Differential heatmap of 13 m6A regulator genes in breast cancer. Red indicates upregulation, and green indicates downregulation. Blue represents normal tissue and red represents breast cancer tissue. *P < 0.05, **P < 0.01, ***P < 0.001. (**B**) A violin plot of differential expression of 13 m6A regulator genes in breast cancer. Blue represents normal tissue and red represents breast cancer tissue. (**C**) Correlation analysis of m6A regulator genes. Red represents positive correlation, blue represents negative correlation. (**D–F**) Identification of consensus clusters by 13 m6A regulator genes. (**D**) The consensus cumulative distribution function (CDF) of the consensus matrix for each K (colored). (**E**) The Delta area diagram shows the relative change of area under the CDF curve. (**F**) The consensus matrix displays the cluster membership marked by colored rectangles, enabling users to calculate the number of cluster members in their consensus context. (**G**) Reliability analysis of tumour consensus clusters. Red represents cluster 1 and blue represents cluster 2. (**H**) Survival analysis of two tumour clusters. Red represents cluster 1 and blue represents cluster 2.

First, we found that 13 m6A regulators, except ZC3H13, were differentially expressed when comparing BC and adjacent normal tissue (Fig. 2A,B). Next, the correlation between m6A regulators was analysed (Fig. 2C). METL3 and HNRNPC had the highest positive correlation, while METL3 and RBM15 had the highest negative correlation, which were 0.5 and −0.44, respectively. To further explore the characterisation of m6A modifications in different clinical phenotypes and their biological consequences, we focused in transcriptome data from BC patients and their comprehensive clinical annotations. Next, we used the unsupervised clustering principle to classify our tumour samples according to m6A regulator expression levels. As can be seen from Fig. 2D,E, when K classification is equal to 4, the growth rate of the cumulative distribution function is slow. However, since the correlation between groups was too high for analysis when the K value was 3, we chose to classify tumours into two types, as shown in Fig. 2F. We conducted principal component analysis (PCA) and survival analysis for the two tumour types. All regulator expression levels were utilised to draw the PCA diagram to judge the effectiveness of our model, cluster 1 and 2 have obvious differentiation, indicating that the results are correct (Fig. 2G). Although Kaplan–Meier analysis showed a p-value of 0.381 between the two groups, there seems to be a certain distance between the 5–20 year survival curves of the two groups, which is worth further attention and exploration (Fig. 2H).

### TME and immune cell infiltration characteristics with different m6A modifications

**Figure 3:**
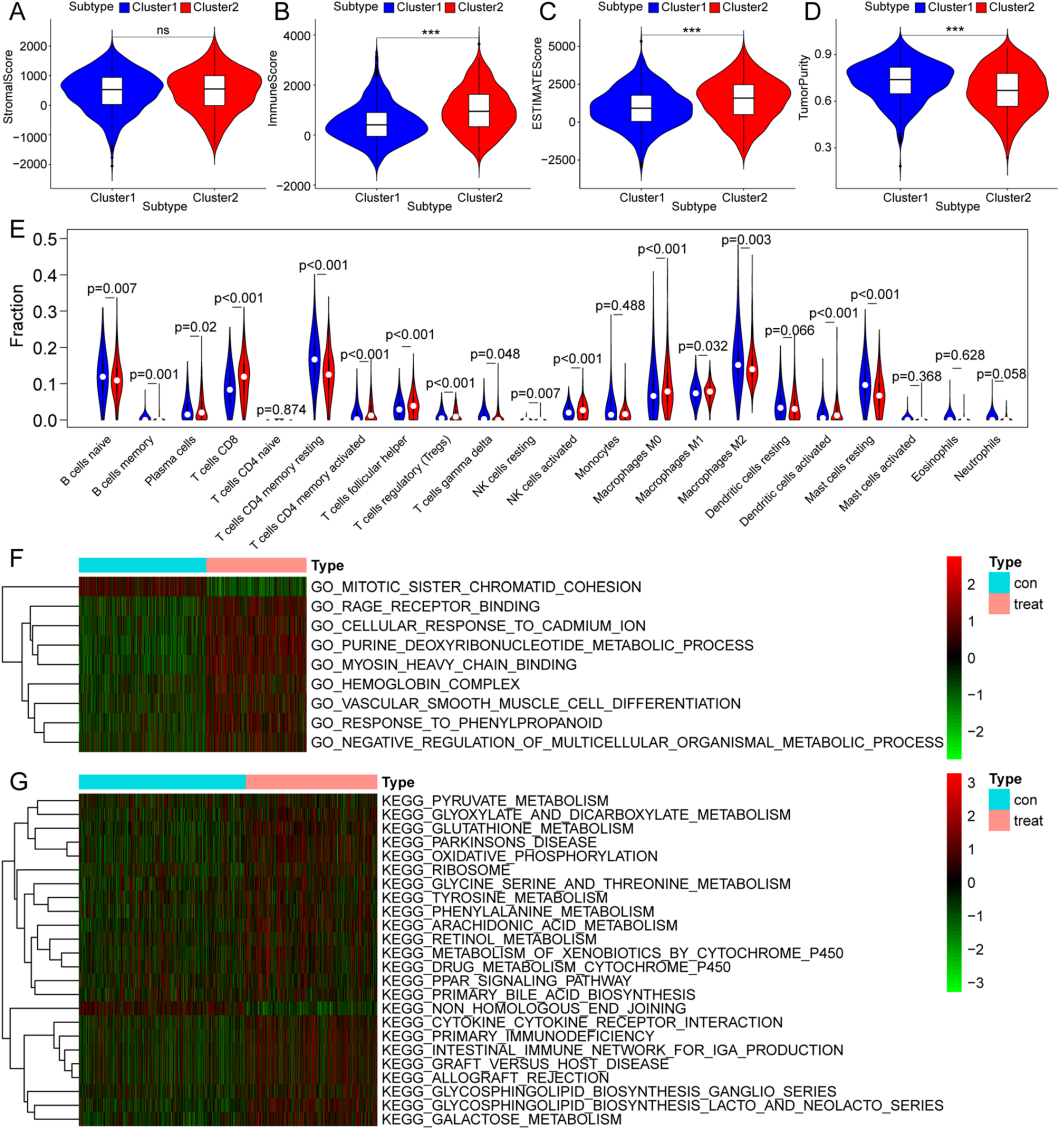
Analysis of tumour microenvironment, immune cell infiltration, and GSVA among different tumour classification. Blue is cluster 1 and red is cluster 2. (**A**–**D**) The relationship between tumour classification and tumour microenvironment. (**E**) The relationship between tumour classification and immune cell infiltration. (**F**,**G**) The relationship between tumour classification and GSVA enrichment. (**F**) Gene ontology analysis. (**G**) Kyoto Encyclopedia of Genes and Genomes pathway enrichment analysis.

To study the invasion characteristics of the TME and immune cells in different tumour groups, we first scored each BC sample using the ESTIMATE algorithm, including a stromal score, immune score, estimate score, and tumour purity, which were shown in different clusters (Fig. 3A–D). A higher score signified greater cell content. Cluster 1 was classified as an immune-inactivated phenotype, which was characterised by immunosuppression and high tumour purity (Fig. 3A–D). On the other hand, cluster 2 had an immune-activated phenotype, characterised by high infiltration of innate immune cells. The difference analysis following the CIBERSORT algorithm showed that activation of adaptive immune cells, including plasma cells, CD8 + T cells, CD4 + memory T cells, T follicular helper cells, T regulatory cells (Tregs), activated NK cells, M2 macrophages, and activated dendritic cells were highly expressed in cluster 2 (Fig. 3E). Gene set variation analysis (GSVA) was subsequently performed to explore the biological properties of the different clusters. Cluster 1 members were involved in upregulation, including the response to phenylpropanoids and non-homologous end joining repair mechanisms. Cluster 2 upregulated functions were primarily immune-related and included vascular smooth muscle cell differentiation, primary immunodeficiency, intestinal immune networks for IgA production, ribosomes, and graft versus host disease functions. Cluster 2 functions were similar to those in tumours, with the presence of infiltrating B lymphocytes (Fig. 3F,G).

### Construction of m6A regulator-based prognostic signatures

**Figure 4:**
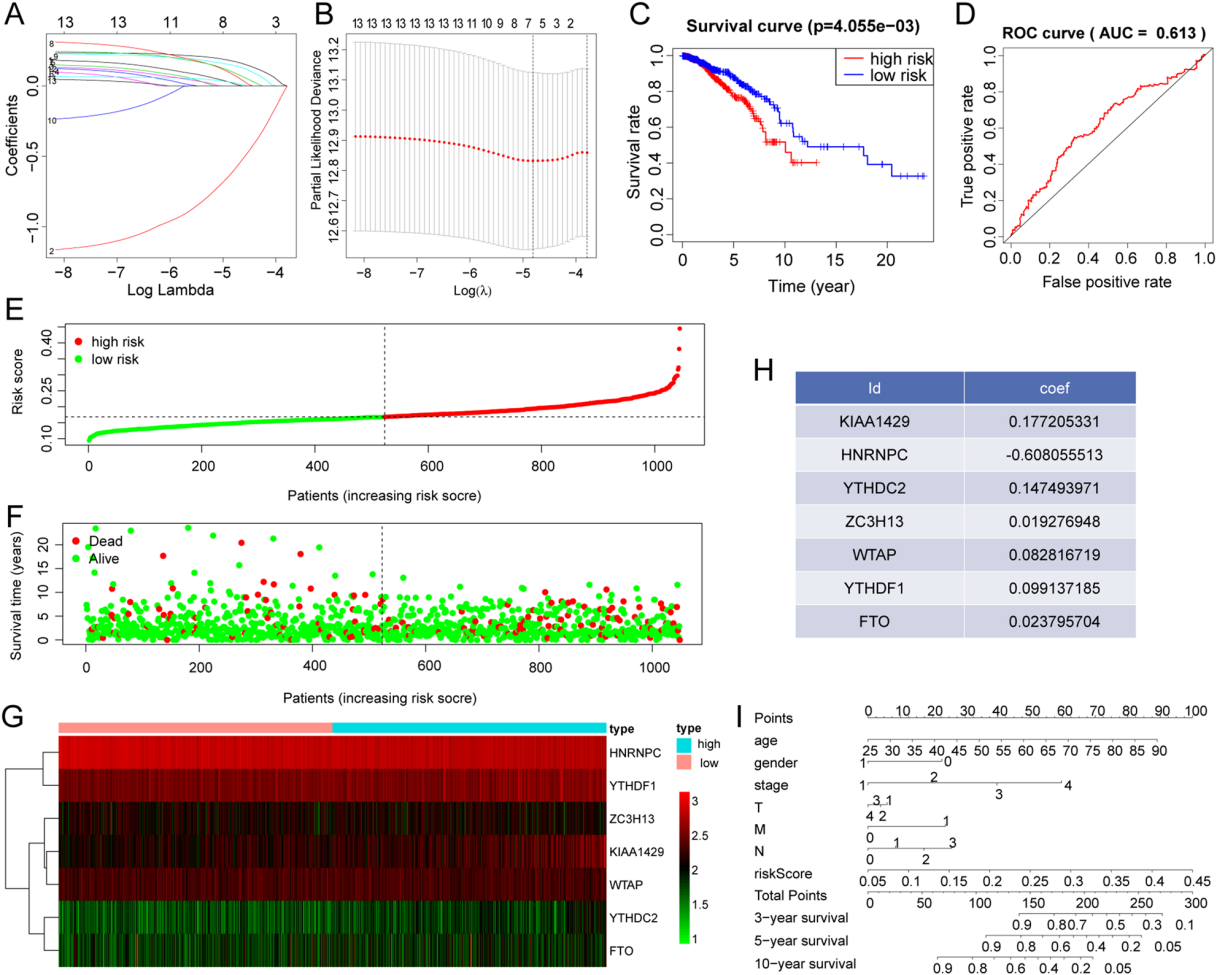
The m6A regulator prognostic model. (**A**,**B**) Identification of prognostic m6A regulator genes via LASSO cox regression analysis. (**A**) The ordinate shows the correlation coefficient, and the abscissa shows the penalty coefficient. (**B**) Partial likelihood bias of the LASSO coefficient distribution. The vertical dashed line indicates the minimum partial likelihood deviation. (**C**) The relationship between risk score and survival in breast cancer patients. Red represent high risk patients and blue represent low risk patients. (**D**) ROC curves showed the predictive efficiency of the risk score in breast cancer. (**E–G**) The distribution of risk score (**E**), patients’ survival status (**F**), and m6A regulators’ expression level (**G**) between high- and low-risk groups. High means high risk, and low means low risk. (**H**) The coefficient of seven prognostic m6A regulators. (**I**) A prognostic nomogram predicting 3-, 5-, and 10-year overall survival with breast cancer.

Univariate and multivariate prognostic analyses were performed for m6A signatures. Univariate prognostic analysis indicates whether the signature is associated with survival, while multivariate independent prognostic analysis can determine the relationship between multiple factors and determine whether a factor of interest can be used as an independent prognostic factor. LASSO analysis can filter factors with high correlation and prevent the model from over-fitting. We used LASSO regression analysis to construct a model to calculate a risk score for each patient (Fig. 4A,B). Each of these patients were allocated into either high-risk or low-risk groups, depending on whether their score was higher or lower than the median risk score. Kaplan–Meier survival curves of patients in the high-risk and low-risk groups showed that patients in the high-risk group had a relatively low survival probability (Fig. 4C). Receiver operating characteristic (ROC) curve analysis was used to assess the prognostic power of the model. The area under the curve (AUC) for the ROC curve was approximately 0.6, indicating a strong prognostic ability (Fig. 4D). The risk score and vital status of each patient in the two groups were visualised using a risk curve, scatter plot, and heatmap (Fig. 4E–G). It shows that with the increased risk score and survival time, an increasing number of patients do not survive. Figure 4G shows the expression of seven m6A regulators in the prognostic model in the high-risk and low-risk patients. The regression coefficients of each m6A regulator in the model are shown in Fig. 4H. To predict the overall survival (OS) of BC patients, multivariate survival analyses were conducted to construct a stable nomogram. In addition to the risk score based on the m6A regulators, clinical factors, including age at initial diagnosis, gender, and pathologic information, such as tumour, nodes, metastases (TNM) stage were included in the nomogram. The TNM stage had the highest weight in the model, with other factors playing a role in model stabilisation. The m6A regulator-based risk score could predict the 3-, 5-, and 10-year survival rates for BC patients, suggesting a strong impact of m6A regulators on patient survival (Fig. 4I).

### Identification of small molecule drugs

**Figure 5:**
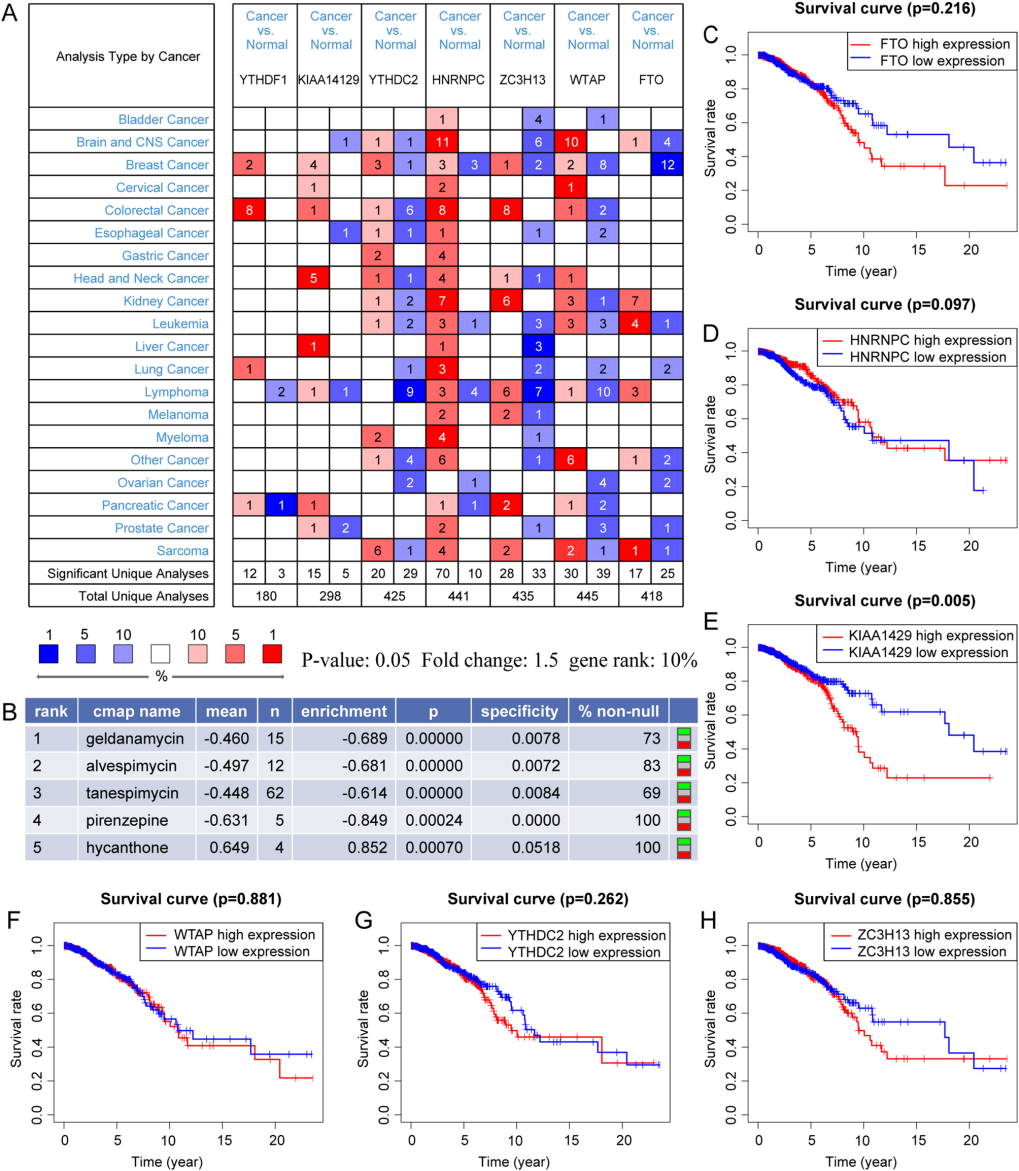
Clinical and pan-cancer characters of m6A regulators. (**A**) The expression of seven prognostic m6A regulator genes of BC in pan-cancer. (**B**) five potential therapeutic drugs predicted by seven m6A regulators in the breast cancer prognostic model. (**C**–**H**) Survival analysis of m6A regulators. (**C**) FTO. (**D**) HNRNPC. (**E**) KICC1429. (**F**) WTAP. (**G**) YTHDC2. (**H**) ZC3H13. Red represents high expression and blue represents low expression.

By performing pan-cancer expression analysis by Oncomine, we discovered that these regulators, which are expressed in other cancers, are similar to those in BC (Fig. 5A). An online website showing connectivity map (CMap)'s research indicated that the expression of seven m6A regulators showed that five small molecules, including geldanamycin, alvespimycin, tanespimycin, pirenzepine, and hycanthone, could be used as potential therapeutic drugs for BC. Figure 5B shows the potential efficacy of these compounds against tumours.

### CNVs and downstream function analysis of m6A regulators

**Figure 6:**
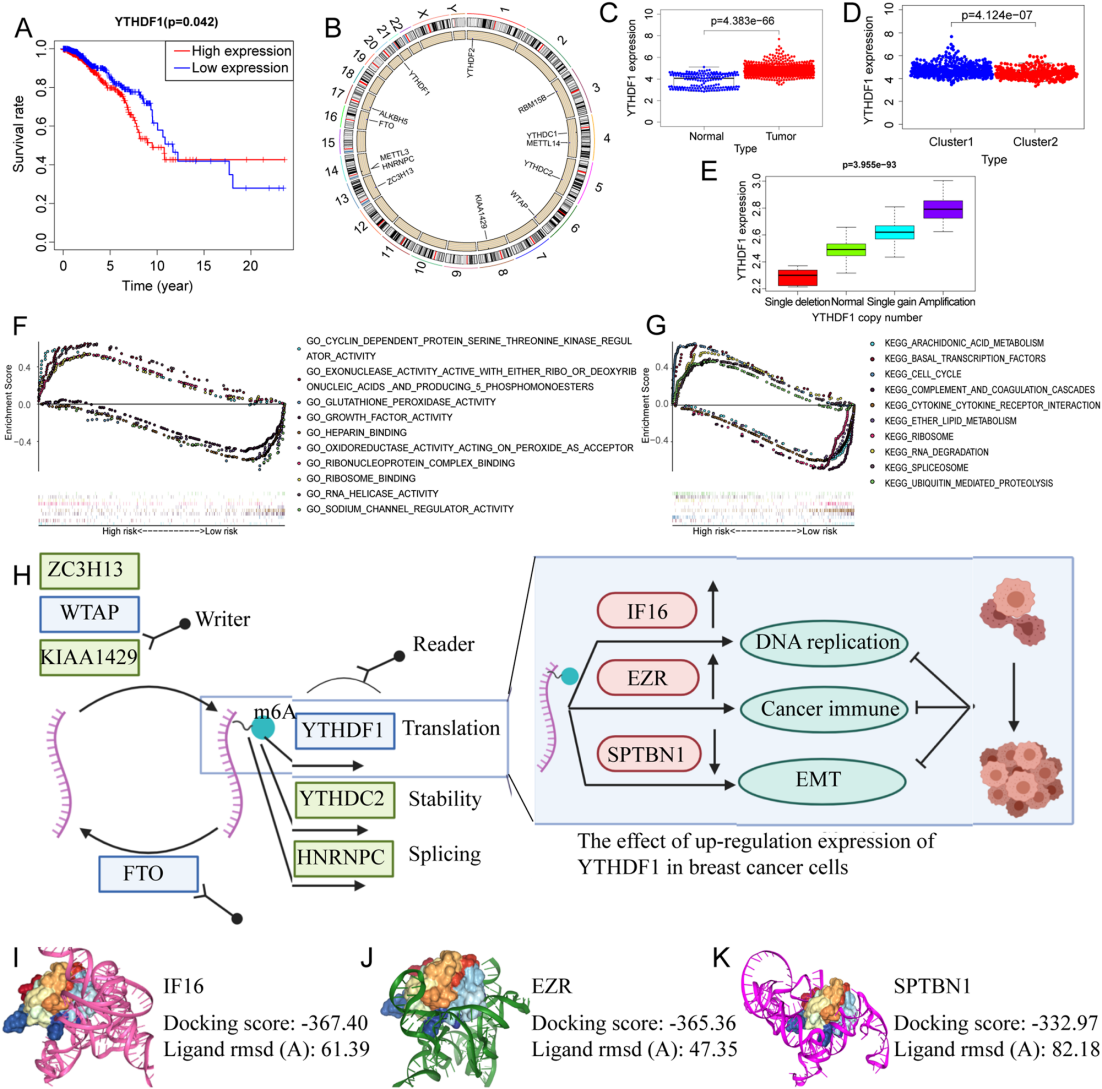
Analysis of the hub gene YTHDF1. (**A**) Survival analysis of YTHDF1. (**B**) Copy number variation analysis of m6A regulators. Black indicates the copy-number amplification, and blue indicates the copy-number lost. (**C**) Difference analysis of YTHDF1 in breast cancer. The blue dots represent paracancer samples, and the red ones represent breast cancer samples. (**D**) The expression of YTHDF1 in the first two clusters. The blue dots represent low immune samples (cluster 1), and the red ones represent high immune samples (cluster 2) (**E**) Copy number variation analysis of YTHDF1. (**F**,**G**) GSEA enrichment analysis. (**F**) Gene ontology analysis. (**G**) Kyoto Encyclopedia of Genes and Genomes pathway enrichment analysis. (**H**) A schematic diagram of m6A regulators and the genes downstream of YTHDF1 in breast cancer. (**I**–**K**) The binding sites of YTHDF1 and its downstream targets were identified. The binding sites of circYap with PABP and eIF4G were predicted by NPDock. The docking quality of the model was confirmed. (**I**) YTHDF1 and IFI6 mRNA. (**J**) YTHDF1 and EZR mRNA. (**K**) YTHDF1 and SPTBN1 mRNA.

Two of the seven m6a-related prognostic regulators (YTHDF1 and KIA1429) had an impact on patient survival (Figs. 5C–H and 6A), with an increasing copy number of both regulators (Fig. 6B). Combined with the differential expression data in BC, the Kolmogorov–Smirnov test showed that YTHDF1 was the hub regulator. To uncover the potential mechanism of YTHDF1 in BC, we first determined the differential expression of YTHDF1 in BC and adjacent normal tissue through a honeycomb diagram and found that YTHDF1 was highly expressed in tumour tissues (Fig. 6C). Subsequently, the expression of YTHDF1 in different clusters was also studied, and we found that YTHDF1 was highly expressed in the immune-inactivated, cluster1 (Fig. 6D). Figure 6B shows the CNV of the m6A regulator on different chromosomes. The inside or outside location of the circle plot shows whether the regulator CNVs are easily lost or obtained. Figure 6E shows the CNV of YTHDF1. We then conducted gene set enrichment analysis (GSEA) and determined that the expression was correlated with the cell cycle, growth factors, and cytokine receptor interactions (Fig. 6F,G). To identify genes downstream from YTHDF1, we reviewed the literature and obtained the upregulated and downregulated sets of genes YTHDF1 knockdown using a study by Liu et al.^18^. These two gene sets intersected with high and low gene expression differences between BC and adjacent normal tissues, respectively. Finally, three genes (IFI6, EZR, and SPTBN1) were identified, among which IFI6 and EZR were upregulated and SPTBN1 was downregulated (Fig. 6H). Molecular docking analysis confirmed that the mRNA of downstream genes could bind with a high degree of precision to the RNA-binding subunit of the YTHDF1 protein (Fig. 6I–K).

### Small conditional RNA data revealed high cell heterogeneity in breast invasive carcinoma

**Figure 7:**
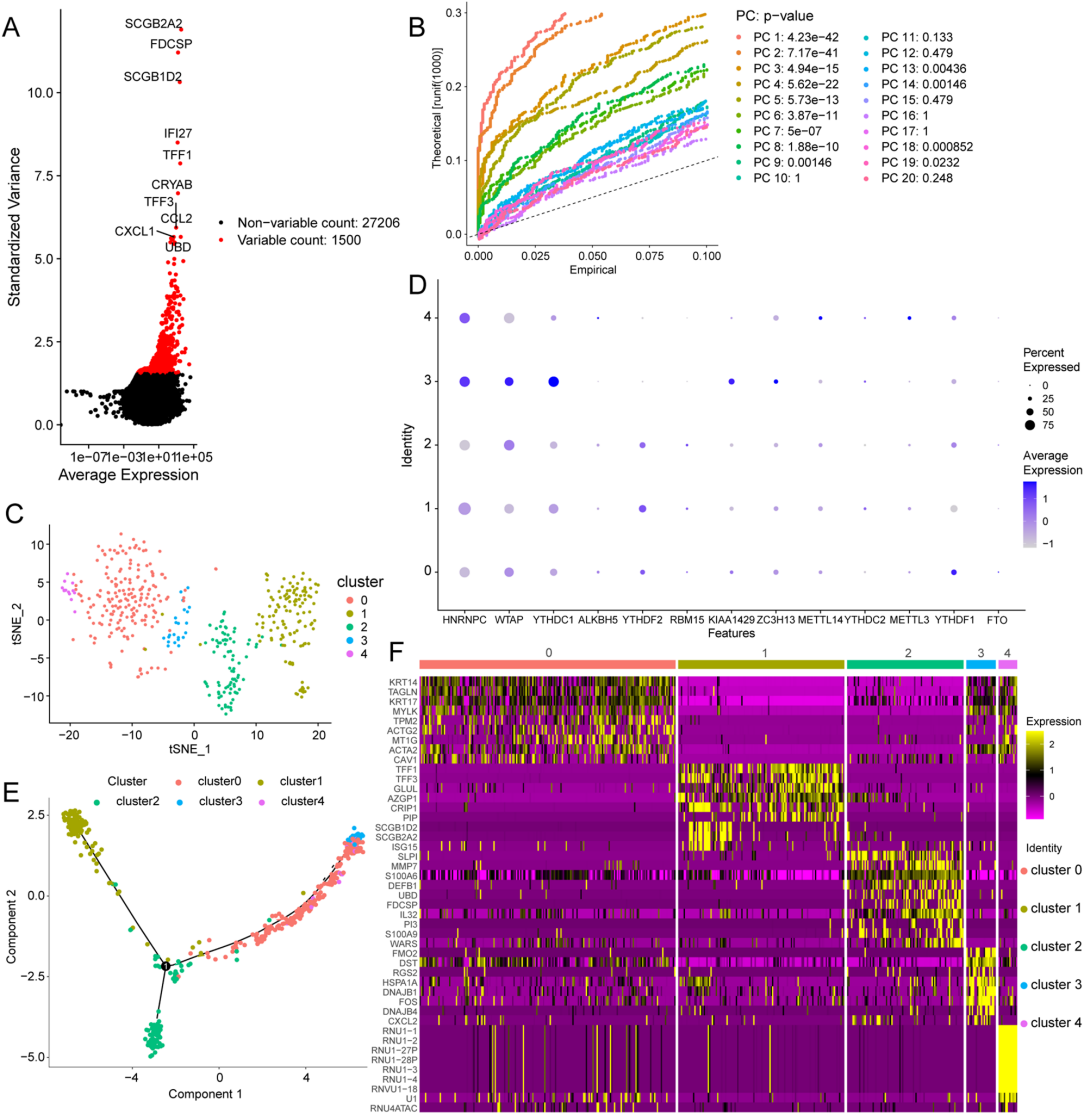
Single-cell sequencing method reveals expression patterns of m6A regulators. (**A,B**) Gene filtering and PCA clustering of gene expression matrix. (**C**) t-SNE projections of IBC tissue. (**D**) Regulation mode of m6A regulators in various invasive breast cancer (IBC) clusters. 0–4 on the Y axis indicate different clusters. (**E**) Cell trajectory analysis of IBC clusters. (**F**) The heatmap shows the marker genes in each cluster.

**Figure 8:**
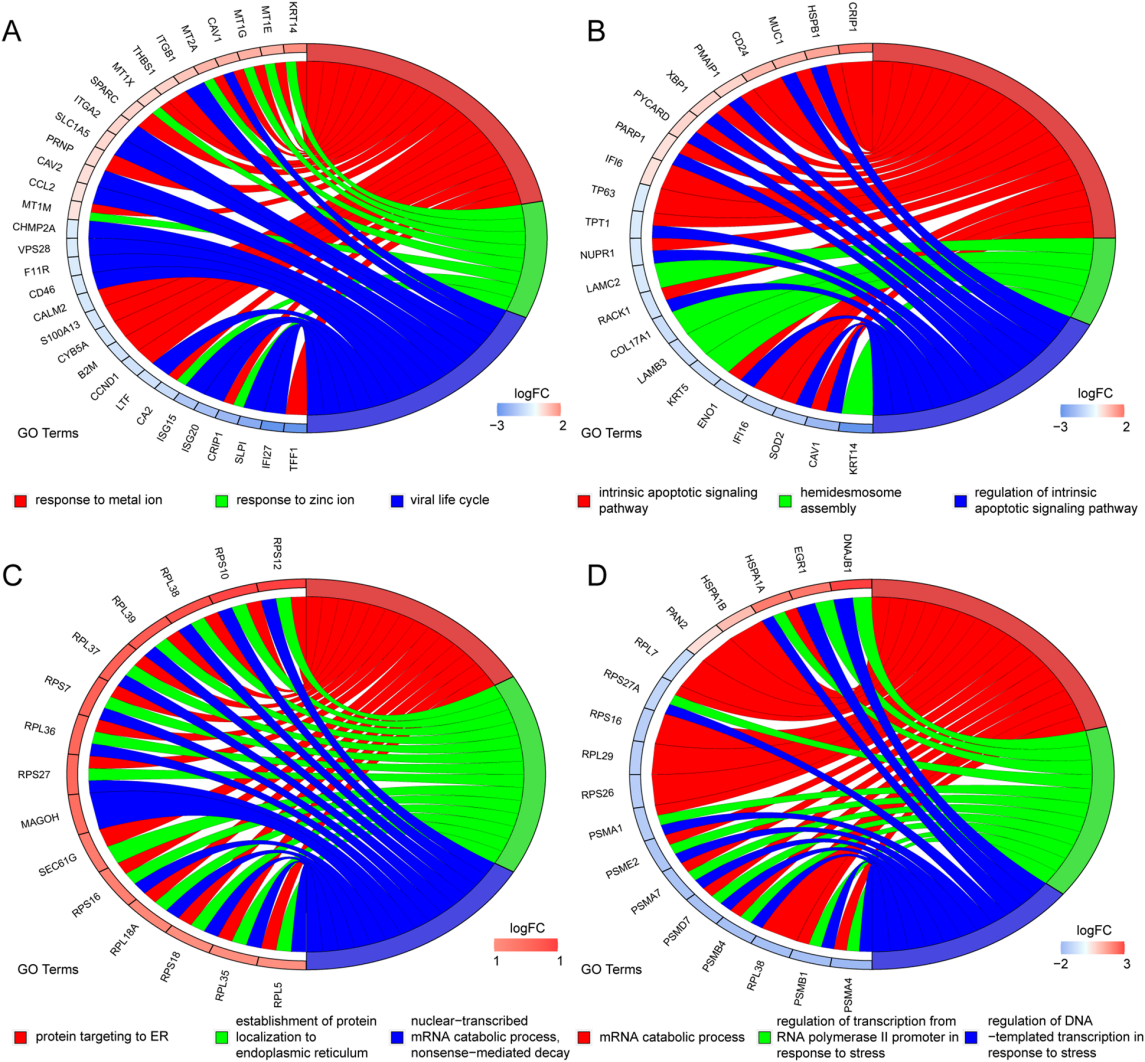
Gene markers and functional analysis of each invasive breast cancer (IBC) cell cluster. The chord plots reveal the highly related functions and genes assigned to IBC cell cluster 0 (**A**), cluster 1 (**B**), cluster 2 (**C**) and cluster 3 (**D**)**.**

To determine intratumor heterogeneity of 13 m6A regulators, expression patterns in BC were studied. First, quality control was performed on the small conditional RNA (scRNA) sequencing data from BC patients (Fig. 7A), and genes with large standard deviations were selected to filter the data. The scRNA-seq data were then normalised, the top 20 principal components were selected, and their p-values and distributions were observed. A p < 0.05 was screened for subsequent analysis (Fig. 7B). The unsupervised analysis of BC cells using t-distributed stochastic neighbour embedding (t-SNE) showed that BC cells were highly heterogeneous and divided into five main clusters (Fig. 7C). We tested the expression patterns of m6A regulators in these cell clusters and found that HNRNPC, WTAP, YTHDC1, KIAA1429, and ZC3H13 were highly expressed in cluster 3, METTL14 and METTL3 were highly expressed in cluster 4, and YTHDF1 was highly expressed in cluster 0 (Fig. 7D). We further used trajectory analysis to evaluate the degree of differentiation of BC cells and found that all cells were projected onto one root and two branches. Interestingly, cells in cluster 2 were mainly located at the root, while cells in cluster 1 were mostly on the left, and cells in clusters 0, 3, and 4 were primarily on the right (Fig. 7E). Next, we tracked the gene markers of these IBC cell groups to determine their molecular characteristics (Figs. 7F and 8). Immune genes, such as SLPI, ISG20, ISG15, LTF, B2M, S100A13, CCL2, were in cluster 0 and immune genes PSMD7, PSME2, HSPA1B, and HSPA1A were identified as specific marker genes in clusters 3. Therefore, we named clusters 0 and 3 ‘immune-related IBC cells’. Autophagy genes, such as TP63 and PARP1, were recognised as specific marker genes in cluster 1. Therefore, we named cluster 1 ‘autophagy-associated IBC cells’. Most of the specific genes in cluster 4 were RNU1 family genes, which have been proven to be involved in the splicing process^19^; therefore, we named cluster 4 ‘splicing-associated IBC cells’. The trajectory analysis results showed that the cells in cluster 2 were mainly located in the root. Therefore, we named cluster 2 ‘stem-like IBC cells’.

### Quantitative reverse transcription real-time polymerase chain reaction to validate regulator expression

**Figure 9:**
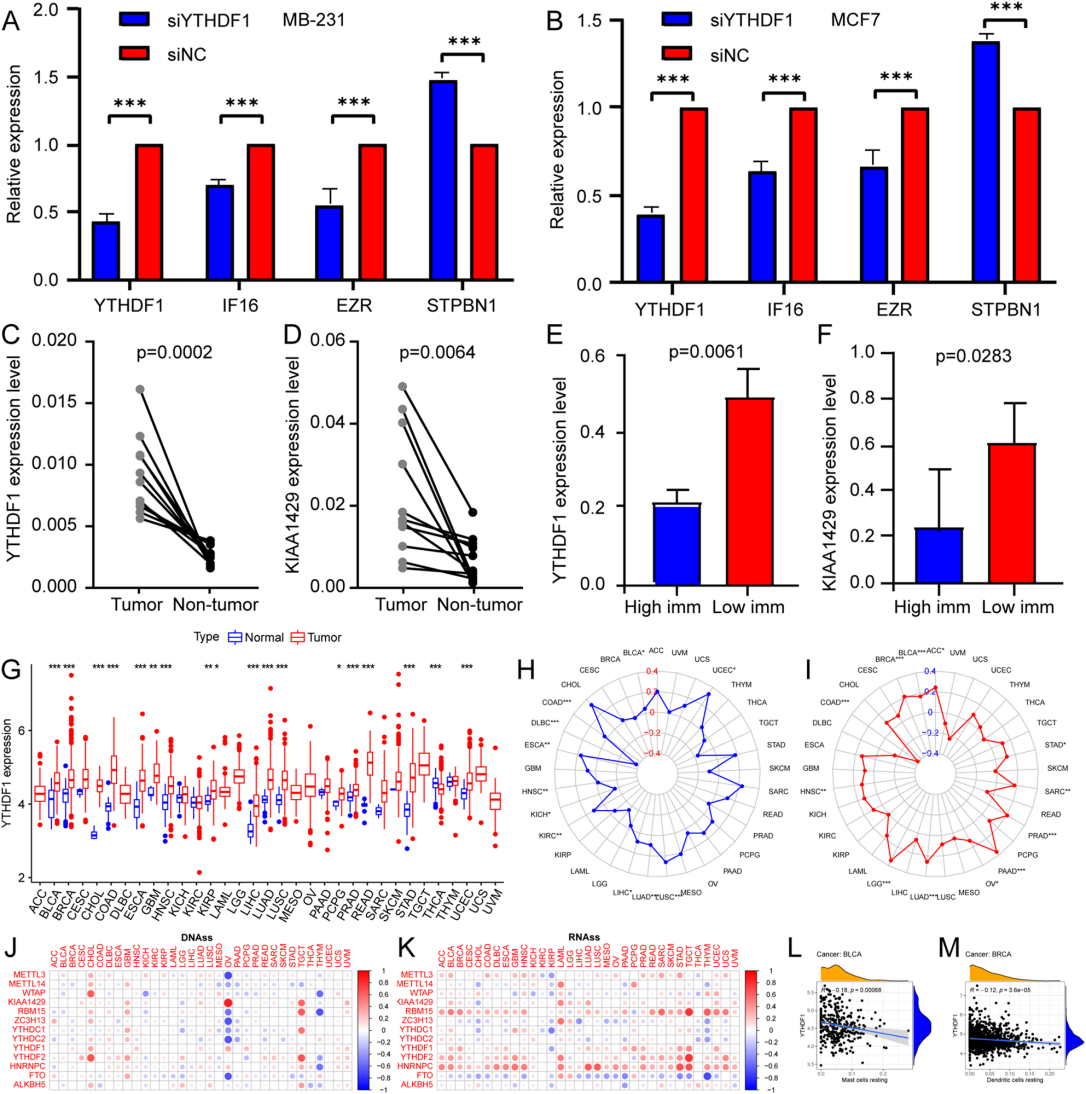
YTHDF1 functional verification. (**A,B**) Expression levels of YTHDF1 and its targets after YTHDF1 knockdown. (**C,D**) YTHDF1 and KIAA1429 expression levels in BC and adjacent breast tissue; (**E,F**) YTHDF1 and KIAA1429 expression levels in high and low immunity group. High imm stands for high immunity group. Low imm stands for low immunity group. (**G**) The expression of YTHDF1 in pan-cancer. Blue represents normal tissue and red represents cancer tissue. (**H,I**) Correlation between YTHDF1 expression and microsatellite instability (**H**) and tumour mutation burden (**I**) in pan-cancer. (**J, K**) Relationship between the DNA (**J**) and RNA (**K**) stemness scores and m6A regulators expression levels in pan-cancer. (**L,M**) Correlation between YTHDF1 expression and immune cell infiltration in pan-cancer. (**L**) Mast cells resting in bladder urothelial carcinoma. (**M**) Dendritic cells resting in breast invasive carcinoma.

To verify the regulatory effect of YTHDF1 on downstream genes, we knocked down YTHDF1 with small interfering RNA (siRNA) in MB-231 and MCF7 cells and found that FI6 and EZR were downregulated and SPTBN1 was upregulated in knockdown cells (Fig. 9A,B). Finally, the expression of YTHDF1 and KIAA1429 in the different groups was confirmed by quantitative reverse transcription real-time polymerase chain reaction (qRT-PCR). Expression of YTHDF1 and KIAA1429 in the BC tissue and the low immunity group (5 cases) was significantly higher than in the adjacent normal breast tissue and the high immunity group (6 cases) (Fig. 9C–F).

### Immune function of YTHDF1 in pan-cancer

**Figure 10:**
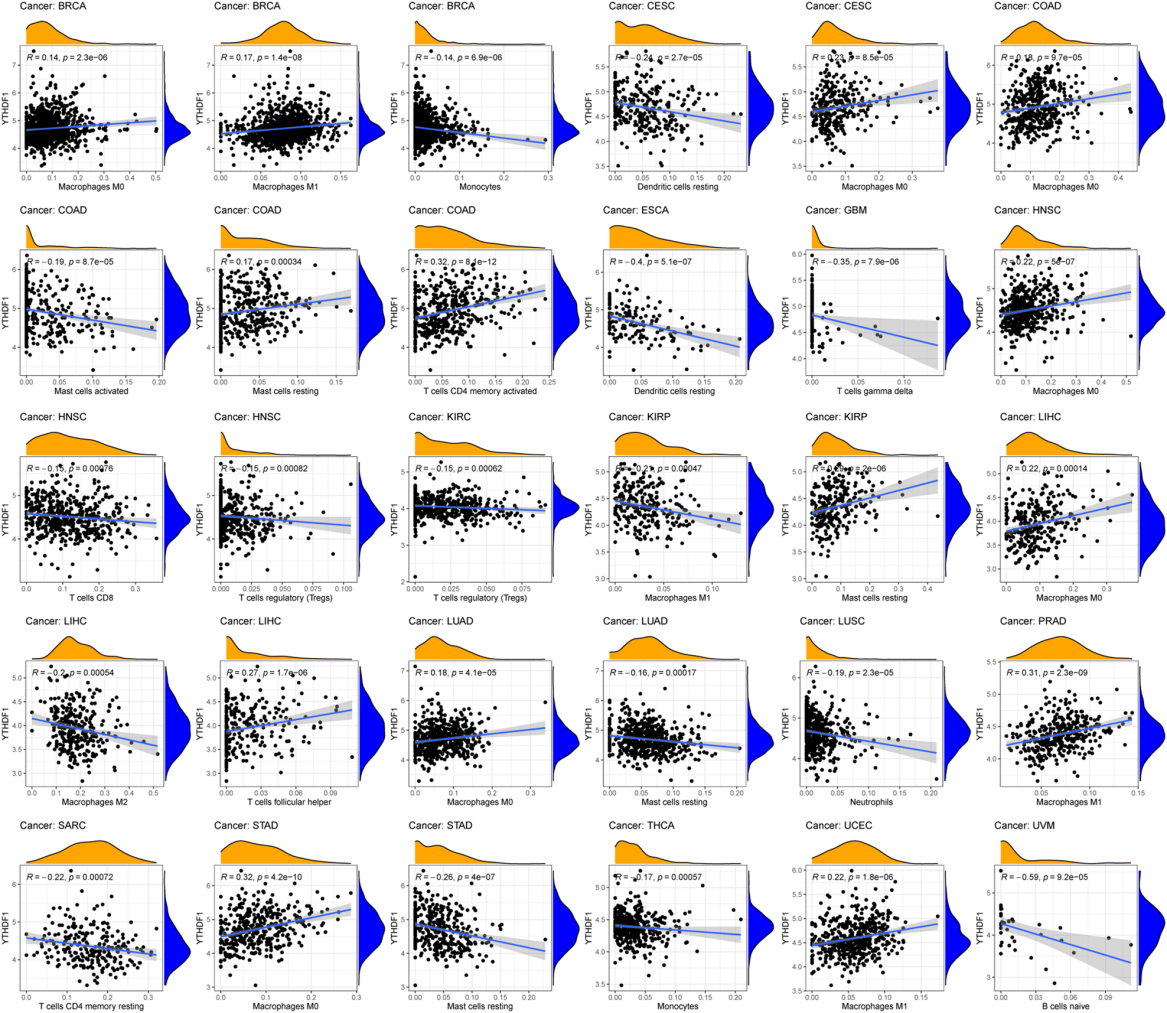
Correlation between YTHDF1 expression and immune cell infiltration in pan-cancer.

**Figure 11:**
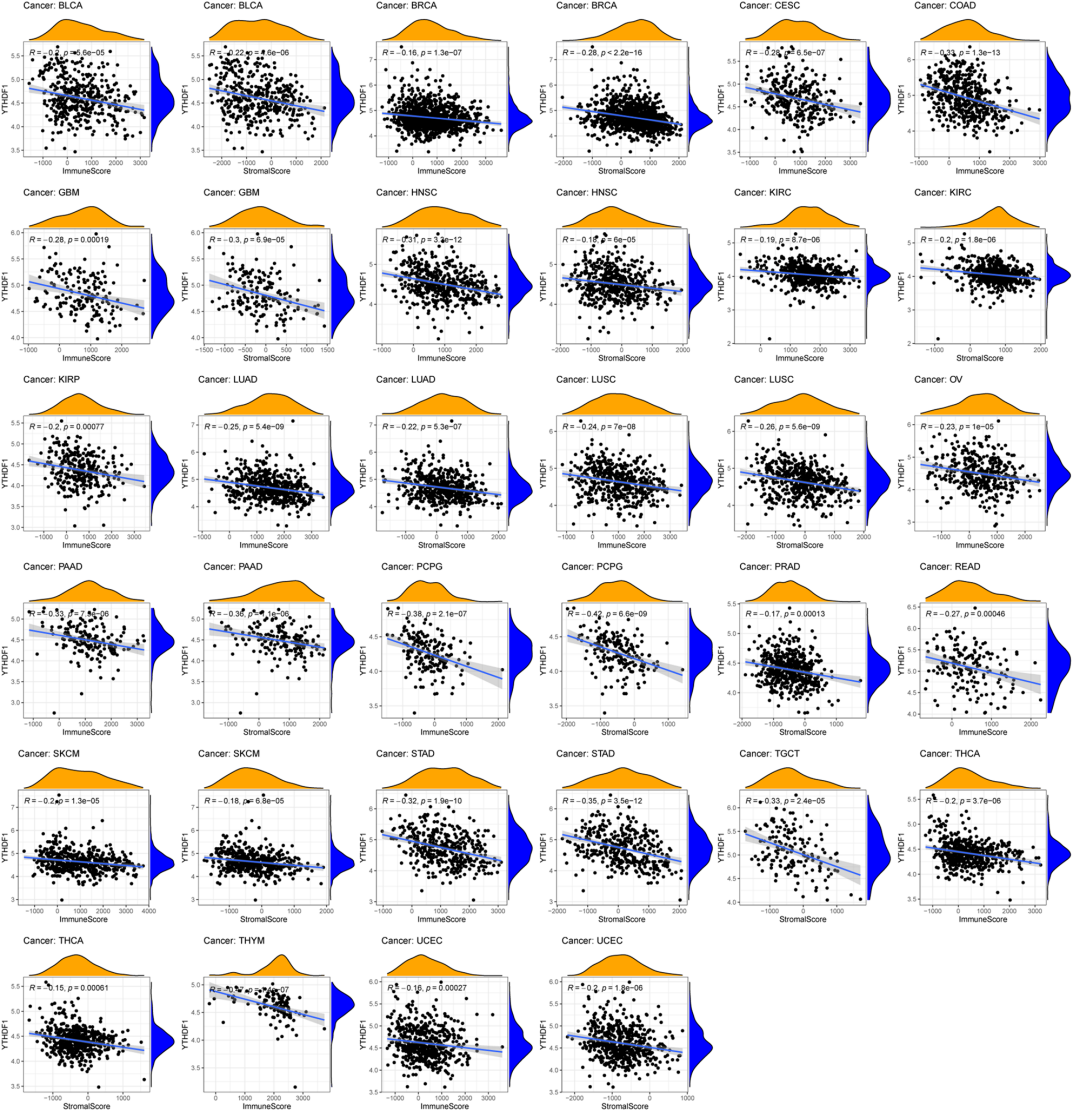
Correlation between YTHDF1 expression and immune and stromal score in pan-cancer.

To further elucidate the function of YTHDF1, we performed pan-cancer analyses of YTHDF1. As shown in Fig. 9G, YTHDF1 was differentially expressed in more than half the cancers when comparing tumours to adjacent normal tissue. Figure 9H,I show the correlation between YTHDF1 and microsatellite instability (MSI) or YTHDF1 and tumour mutational burden (TMB) in pan-cancer analysis, respectively. YTHDF1 was positively correlated with TMB in adrenocortical carcinoma, stomach adenocarcinoma, sarcoma, prostate adenocarcinoma, pancreatic adenocarcinoma, ovarian serous cystadenocarcinoma, lung adenocarcinoma (LUAD), brain lower-grade glioma, head and neck squamous cell carcinoma (HNSC), breast invasive carcinoma (BRCA), and bladder urothelial carcinoma (BLCA), while it was negatively correlated with TMB in colon adenocarcinoma (COAD). YTHDF1 was positively correlated with MSI in BLCA, uterine corpus endometrial carcinoma, lung squamous cell carcinoma, LUAD, liver hepatocellular carcinoma, kidney renal clear cell carcinoma, esophageal carcinoma, and COAD, but it was negatively correlated with MSI in kidney chromophobe, HNSC, and lymphoid neoplasm diffuse large B-cell lymphoma. YTHDF1 was positively correlated with the DNA and RNA stemness scores in most cancers (Fig. 9J, K). We found that YTHDF1 was significantly correlated with immune cell infiltration in 18 cancers, involving 15 immune cell types, among which macrophages were correlated with 11 cancers and mast cell resting were correlated with five cancers (Figs. 9L,M and 10). Figure 11 shown the correlation between YTHDF1 and the TME in pan-cancer. We can seen that YTHDF1 was related to the TME of 21 cancers, the immune score of 19 cancers, and the stromal score of 15 cancers.

## Discussion

The expression analysis of m6A regulators and their clinical characteristics revealed that m6A methylation regulators play a crucial role in BC prognosis^20,21^. Recent evidence suggests that m6A modification is involved in the regulation of multiple tumour progression processes, including tumourigenesis, proliferation, differentiation, and metastasis^22,23^. Previous studies have shown that the m6A-modified genes perform a carcinogenic role in some instances, such as in colorectal cancer where the overexpression of METTL3 leads to higher m6A levels of the oncogene SOX2^24^. The m6A regulator IGF2BP2 is also up-regulated and enhances the stability of target genes, such as DANCR, and thus promotes stemness-like properties and cell proliferation of tumours^25^. On the contrary, m6A can have a cancer-suppressing role. The knockdown of METL14 abolishes m6A levels of the downstream target, the lncRNA for XIST, and augments XIST expression, thus promoting the invasive ability of colorectal cancer cells and tumourigenicity^26^. Potential strategies for cancer treatments can be leveraged through knowledge of m6A modifications. Advances include the use of meclofenamic acid 2, also known as the ethyl ester derivative of meclofenamic acid, which acts as a selective FTO inhibitor by competing for FTO binding sites, suppressing tumour progression in glioblastoma^27^. In our study, we identified seven regulators associated with BC prognosis. Subsequent pan-cancer studies of these genes showed that the expression of these regulators in multiple cancers was similar to that in BC. Further, small molecule compounds were screened and analysed to obtain potential therapeutic drugs for BC, among which geldanamycin, doxorubicin geldanamycin, alvespimycin, and tanespimycin were considered to be strong Hsp90 inhibitors and have proven to be important therapeutic targets for inhibiting the proliferation of BC cells^28–30^.

While the role of the m6A modification in antitumour immunity is crucial, the overall characterisation of the BC immune infiltrate is affected by multiple m6A regulators and these are not fully understood since most studies focus on a single TME cell type or a single regulator^20,21,23^. According to Zhang et al., three m6A modification patterns were identified in gastric cancer and these are characterised as the immune-excluded phenotype, the immune-inflamed phenotype, and the immune-desert phenotype^16^. Identifying the TME cell infiltration characteristics under distinct m6A modification patterns may enhance the understanding of the TME and antitumour immune responses. In our study, two distinct patterns, including immune activity and inactivity were discovered. ESTIMATE analysis showed that tumour tissue in the immune-active group has a lower tumour purity and a higher infiltration of immune cells. A significant difference between immune cell infiltrates was observed in separate cluster groups when applying the CIBERSORT algorithm. In the immune-active group, activated T- cells and NK cells were up-regulated while M2 macrophages were down-regulated. Some scholars have confirmed that the T cell immune response is paramount in the antitumour immune response, with the number of infiltrating T cells in the TME correlating with improved patient survival^31,32^. Activation of NK cells and deactivation of mast cells are considered to be a response to tumour-derived metabolites^33^, such as oxidised natural polyamines^34^. Tumour-associated macrophages (TAM), a main component of the BC TME, exhibit a crucial role in reconstructing the tumour extracellular matrix, vascular remodelling, recruiting immunosuppressive leukocytes, and evading the host immune system by responding to various signals. Depending on signals in the microenvironment, macrophages can be polarised into different subtypes, including classically activated (M1) and selectively activated (M2) macrophages, in which M2 polarisation is similar to TAM^35^. Clinicopathological evidence suggests that the accumulation of TAM, especially M2, is related to poor patient survival^36^. We observed that the immune-activated cluster 2 had relatively activated innate and adaptive immunity and better survival. In addition, GSVA analysis revealed that the biological pathways with differential activation included angiogenesis, cytokine receptors, IgA, and various metabolism factors and these were under different m6A modification modes, indicating that m6A significantly promotes tumour progression through multiple mechanisms, including the immune system.

To strengthen the understanding of BC heterogeneity, genetic assays have been gradually combined with clinical and pathological features, which allows a greater understanding and classification of BC subtypes, leading to improved prognosis and treatment^4,37^. At the same time, this information provides support for the improvement of existing BC detection tests (The Breast Cancer Index SM, Oncotype DX®kits, and MammaPrint™)^38–40^. We obtained the risk prediction model for BC through LASSO regression analysis and identified seven marker regulators in different survival periods for BC patients, in which KIAA1429, WYAP, and ZC3H13 were classified as ‘writer’, FTO was classified as ‘eraser’, and HNRNPC, YTHDC2, and YTHDF1 were classified as ‘reader’ in relationship to their m6A modifications. The results of the ROC curve analysis showed the prognostic value of the model. Survival analysis showed that KIAA1429 and YTHDF1 were survival-related prognostic regulators. Single-cell analysis showed that HNRNPC, WTAP, KIAA1429 and YTHDF1 were highly expressed in immune-related IBC cells. Previous studies have shown that HNRNPC may be associated with immune response in endometriosis^41^, WTAP is associated with T cell-related immune response in gastric cancer^42^, KIAA1429 can be used as a potential immunotherapy target for LUSC^43^.

CNVs were associated with various clinicopathological factors in patients with cancer and have emerged as one of the major contributors that drive aberrant expression of oncogenes and regulate cancer progression^44^. However, few CNV-related gene signatures have been identified in cancer since copy number segments are unrelated to gene length^45^. To discover the potential targets of BC, we intersected the prognostic regulators with the CNV-related differential genes and identified a hub regulator, YTHDF1, which is highly expressed in BC and cluster1 (immune inactivated cluster), and is classified as a ‘reader’ that recognises, binds, and promotes the translation efficiency of m6A^17,21^. Previous studies have reported that YTHDF1 overexpression commonly occurs in various tumour types^46^. It also plays a crucial role in stem cell-like activity in colorectal cancer cells^47^. In this study, GSEA results show that YTHDF1 expression is highly concerned with cell cycle regulation, RNA degradation, and cytokine receptor interaction. Then, through literature review and a series of analyses, we obtained three potential downstream targets of YTHDF1 in BC, including IFI6, EZR, and SPTBN1. A program was used to verify molecular docking between the tertiary structure of the YTHDF1 protein and the nucleic acid tertiary structure of its downstream genes. The YTHDF1 protein binds to and regulates the mRNA of these three downstream genes. EZR, also known as Ezrin, belongs to the ERM protein family and serves as an intermediate between the actin cytoskeleton and the plasma membrane. It also plays an important role in the adhesion and migration of the tumour cell surface structure^48^. EZR has been shown to promote tumour progression by modulating the nuclear translocation of YAP in pancreatic cancer and by overexpression in glioblastoma by inactivating the NF2 tumour suppressor, which leads to tumour growth^49,50^. Overexpressed IFI6 produces metastatic potential by inducing a redox imbalance in mitochondria and causes BC cells to become resistant to tamoxifen^51^, while silencing IFI6 leads to abnormal apoptosis and growth retardation of BC cells^52^. SPTBN1 is a tumour suppressor gene in many kinds of tumours^53^. The loss of SPTBN1 activates Wnt signalling and promotes the acquisition of the stemness feature in tumour cells, ultimately leading to the progression of malignant tumours^54^. Reviewing the literature, we found that SPTBN1 was downregulated in breast cancer cells and was a key regulator that inhibits EMT and breast cancer growth^55^. This study found that YTHDF1 promotes EMT and breast cancer progression in breast cancer. The regulatory effects of SPTBN1 and YTHDF1 in breast cancer are opposite, which is consistent with the reverse regulation of YTHDF1 by SPTBN1 in this study, and the article by Liu et al.^18^ also mentioned that SPTBN1 reversely regulates YTHDF1.

YTHDF1 was knocked down by siRNA, the expressions of IFI6 and EZR were down-regulated, and the expression of SPTBN1 was up-regulated. PCR results of cancer and adjacent paracancerous tissues showed that KIAA1429 and YTHDF1 were highly expressed in cancer tissues, which was consistent with our bioinformatics results. Secondly, the PCR results also showed that the two regulators were low expressed in the high immune group. Combined with the expression results of cancer and paracancerous tissues, we found that the results were consistent with the results of our bioinformatics method that the tumour tissue of the immunoactive group had lower tumour purity and higher immune cell infiltration.

In conclusion, our research shows that m6A regulates the TME through two mechanisms: immune activation and immune inactivation. These findings may guide future immunotherapy strategies. A prognostic model containing seven m6A regulators was established using multivariate analysis. Finally, combined with CNV data, YTHDF1 was identified as the hub m6A regulator. Its expression and function were verified at the molecular biological and pan-cancer levels, providing molecular targets for prognostic evaluation and immunotherapy in BC.

## Methods

### Data collection and differential analysis

Expression data from fragments per kilobase of transcript per million mapped reads (FPKM) and corresponding clinicopathological information from 1104 BC samples and 113 para-cancerous samples were downloaded from The Cancer Genome Atlas (TCGA) database (https://portal.gdc.cancer.gov/). Breast sample expression data from 80 healthy individuals were downloaded from the Genotype-Tissue Expression database (https://commonfund.nih.gov/GTEx/) and applied as para-cancerous supplementary data. The Perl and R programming languages were used to acquire the intersection between the genes in the two databases, and their expression was corrected in batches. CNV data for BC samples were downloaded from the TCGA database. The m6A regulators were obtained from Zhuang et al.^56^, and an expression matrix of these 13 regulators was extracted from the combined expression data using the Perl language.

The Wilcoxon test was used to analyse the differences between the 13 m6A-related regulators, with the screening condition set at p < 0.05. The R packages ‘pheatmap’, ‘vioplot’, and ‘corrplot’ were used to visualise these regulators differences.

### Regulators of m6A based on unsupervised clustering

BC patients were divided into different groups by performing cluster classification in the R package ‘ConsensusClusterPlus’ (http://www.bioconductor.org/packages/release/bioc/html/ConsensusClusterPlus.html) to identify the expression of m6A regulators. The program was set to repeat 50 times (reps = 50), with an 80% resampling rate (pItem = 0.8). To determine the effectiveness of grouping, we performed PCA on the expression of all genes. OS between the different groups was compared using the Kaplan–Meier method.

### Estimation of TME cell infiltration and gene set variation analysis (GSVA)

To observe the TME and immune cell infiltration in BC patients, we first used the ‘Estimation of STromal and Immune cells in MAlignant Tumours using Expression data’ (ESTIMATE) algorithm to calculate the immune score, stromal score, ESTIMATE score, and tumour purity of each patient. This algorithm uses gene expression signatures to infer the ratio of stromal and immune cells in tumour samples^57^. Then, the CIBERSORT deconvolution algorithm was used to quantify the relative scores of 22 immune cell types based on the transcriptome data. The Wilcoxon test was used to compare the differences between the two groups that were clustered and classified by the expression of the m6A regulator. The immune cells investigated included naïve B cells, memory B cells, plasma cells, CD8 + T cells, naïve CD4 + T cells, resting memory CD4 + T cells, activated memory CD4 + T cells, T follicular helper cells, Tregs, gamma delta T cells, resting NK cells, activated NK cells, monocytes, M0 macrophages, M1 macrophages, M2 macrophages, resting dendritic cells, activated dendritic cells, resting mast cells, activated mast cells, eosinophils, and neutrophils. The variation in biological process activity of different m6A modification patterns was uncovered through GSVA enrichment analysis, a non-parametric method. The GSVA analysis was performed in R language, and the screening condition was adjusted to p < 0.05. The results were visualised using the ‘pheatmap’ package.

### Prognostic model construction

LASSO regression analysis of m6A regulators was conducted by the R ‘glmnet’ package, which selects powerful prognostic predictors by ensuring that the regression coefficient is less than a constant value, avoiding model over-fitting. Samples were divided into high-risk and low-risk groups based on the median risk score. Subsequently, we observed the OS of these two groups using the ‘survival’ package in R. To determine model stability, ROC curve analysis was performed using the ‘survivalROC’ package, and the AUC was calculated. The risk curve and scatter plot were drawn to show the risk score and survival status of each patient, and the heat map demonstrated the expression of different regulators in each risk group. A nomogram predicting 3-, 5-, and 10-year OS of patients was constructed by combining clinical characteristics and risk factors generated by the m6A regulators prognostic model using the ‘rms’ package in R.

### Prognostic regulators analysis

The Oncomine pan-cancer cell-free assay (http://www.oncomine.org) is one of the largest oncogene chip databases that integrates over 715 gene expression datasets of 86,733 cancer and normal tissue samples. It contains comprehensive information on cancer mutation profiles, gene expression data, and relevant clinical features. Oncomine was used to detect differential expression of regulators between BC and normal tissues and among various tumour types. To screen for small-molecule drugs that target m6A regulators which may have a potential therapeutic effect in BC, the CMap database was employed. A list of m6A regulators, including their regulatory details, was uploaded to CMap (https://portals.broadinstitute.org/CMap/) to identify related molecular agents. The CMap database combines gene expression profiles with the disease-specific gene signatures to determine a possible link between drug molecules, gene expression profile data, and diseases. To determine the prognostic value of these regulators in BC, survival analysis was performed, and screening for survival-related m6A regulators was conducted, with p < 0.05.

### Screening and analysis of hub regulator

CNV data were processed using the Perl programming language, and the chi-square test was performed in R language to screen out CNV-related differential genes, and the screening condition was adjusted to p < 0.05. CNV-related differential genes were combined with the expression matrix of differential expression data using logFC = 0.5 and p < 0.05. The hub regulators were acquired by intersecting these genes with m6A regulators related to survival.

To determine the expression of the hub regulators in BC and different cluster groups, such as low versus high immunity, the ‘limma’ package (http://www.bioconductor.org/packages/release/bioc/html/limma.html) was used, and results visualisation was performed with the ‘beeswarm’ package. The Kolmogorov–Smirnov test was used as a normality test to evaluate the CNV of the hub regulators and gene expression levels.

### GSEA and molecular docking

To study the potential biological functions of the hub regulators, we divided the samples into two groups according to the hub regulator gene expression. GSEA software (version 4.0.3) was used to perform Gene Ontology and Kyoto Encyclopaedia of Genes and Genomes enrichment analyses under the condition FDR < 0.05. To further study YTHDF1, we first obtained the gene sets that were upregulated and downregulated after YTHDF1 knockdown through a literature review^18^. Data were then screened to identify genes that are differentially expressed in BC using differential analysis. The two gene sets, high and low expressed genes, were intersected to screen out the genes downstream of YTHDF1, which included IFI6, EZR, and SPTBN1. The online program RNAfold (http://rna.tbi.univie.ac.at/cgi-bin/RNAWebSuite/RNAfold.cgi) was used to form the secondary structure of IFI6, EZR, and SPTBN1 and the mRNA tertiary structures were formed by the online tool, RNAComposer (http://rnacomposer.ibch.poznan.pl/). The crystal structure of the YTHDF1 (binding site complex, PDB ID: 4RCJ) was acquired from the Protein Data Bank and the nucleic acid-protein structures were predicted and visualised by HDOCK (http://hdock.phys.hust.edu.cn/), which was performed using a hybrid docking algorithm that employs template-based modelling by applying protein-RNA benchmarks.

### Single-cell analysis

We obtained single-cell RNA sequencing datasets of BRCA tissue from the Gene Expression Omnibus database (GSE138536). The Seurat package in R3.6.2 was used to filter, reduce dimension, and cluster the data. SingleR identified cell types, while Monocle analysed cell differentiation trajectory.

### Patients

We collected BC samples and adjacent, normal para-cancerous samples from 11 patients who underwent modified radical mastectomy after four rounds of neoadjuvant chemotherapy, including docetaxel combined with epirubicin and cyclophosphamide (TEC), in the Department of Endocrinology and Breast Surgery of the First Affiliated Hospital of Chongqing Medical University from October 2019 to May 2020. The inclusion criteria were as follows: (1) patients with pathological diagnosis of triple-negative BC; (2) patients without distal metastases; (3) patients with TNM stage I-II; (4) patients whose host quality of immune function was detected as high or low immunity group three days before the operation. The resected tissue were frozen in liquid nitrogen. The test results and groups are shown in Table 1. This research was approved by the ethics committee of Affiliated Hospital of Chongqing Medical University (2020-155). All patients were informed and provided with a written informed consent form.

**Table 1:** Results and groups of host quality of immune function.

Item	Patient 1	Patient 2	Patient 3	Patient 4	Patient 5	Patient 6	Patient 7	Patient 8	Patient 9	Patient 10	Patient 11	Reference range
ZLBXB-FZ1	19.55	20.4	16.44	27.48	20.5	39.57	18.22	45.72	32.33	39.03	20.05	27.90–37.3
CD3 +	69.65	85.44	84.45	79.04	73.74	66.35	59.19	66.33	68.86	79.08	75.08	26.00–76.80
CD3 + CD4 + CD8-	35.67	54.32	53.39	43.43	31.6	44.78	42.21	43.01	41.94	48.96	47.14	30–46
CD3 + CD4-CD8 +	28.48	28.21	28.23	33.55	32.53	21.73	15.72	25.39	26.54	27.14	25.51	19.2–33.6
CD4 + CD8 +	0.97	1.64	0.85	1.5	0.5	0.45	0.15	2.87	0.46	0.17	0.23	0–2.00
CD4-CD8-	6.05	4.54	2.78	3.46	11.53	3.69	1.75	1.24	1.6	4.32	2.93	0–12.00
CD3-CD19 +	13.72	3.01	4.34	2.61	4.63	16.71	24.09	16.96	23.42	12.31	14.96	8.50–14.50
CD3-CD16/56 +	16.51	11.55	11.07	18.25	21.57	11.46	15.42	16.71	7.76	7.88	9.96	9.50–23.50
CD3 + CD16/56 +	4.59	7.56	5.04	3.61	7.9	0.49	1.45	4.79	6.96	3.39	1.04	–
Comprehensive score	84	35	52	32	67	375	367	353	693	352	397	–
High or low flag	L	L	L	L	L	H	H	H	H	H	H	–

### Cell culture

The human triple-negative BC cell line, MB-231, and human breast cancer cell line, MCF7, were purchased from ATCC. The cells were cultured in Dulbecco's Modified Eagle's Medium supplemented with 10% FBS and cultured at 37 °C and 5% CO2.

### RNA interference

Early passages of MB-231 and MCF7 cells were transfected with siRNA (25 nM) specific to YTHDF1 (siRNA sequence: cagtggatttttgttaaggatgt, synthesised by Ribobio, Guangzhou, China) using the Lipofectamine RNAiMAX Transfection Reagent (13778-150, ThermoFisher Scientific, IL, USA) according to the manufacturer’s instructions.

### Total RNA extraction

Total RNA was extracted from BC tissue using a UNIQ-10 Column Total RNA Extraction Kit (Sangon Biotech, China). The concentration and purity of RNA were determined using a SMA4000 microspectrophotometer (Merinton Instrument, Inc.) and a DYY-6C electrophoresis apparatus (Liuyi, China).

### qRT-PCR

RNA from human BC tumour tissues was reverse transcribed using RR047A cDNA synthesis kit (TaKaRa, China). 2X SG Fast qPCR Master Mix (High Rox, B639273, BBI) was used for qRT-PCR of KIAA1429 (forward primer, CGATAACTTGATGACCCCAGAA; reverse primer, ATAACGGCAAGATTCCATTTC), IFI6 (forward primer, CTTGTGCTACCTGCTGCTCT; reverse primer, GTTGGAGGCTGCAGTGTACT), EZR (forward primer, ATCAACAAGCGGATCCTGCA; reverse primer, GCTCCTCCTTGGTCTTCACC), SPTBN1 (forward primer, TGAGCAGGCCATCAAGGAAG; reverse primer, CTCGTCGATCCAGAGCTCAC) and YTHDF1 (forward primer, TAAGGAAATCCAATGGACGG; reverse primer, TTTGAGCCCTACCTTACTGGA3) in the StepOnePlus fluorescence quantitative PCR instrument (ABI, Foster, CA, USA). GAPDH (forward primer, GCCCGTTTGCATTTTGTGGAG; reverse primer, CCAACTTTCGGGAA ATCCAT; product length, 126) was used as the internal control gene. T-test and paired t-test were performed using Graphpad Prism 8.0.

### Validation of pan cancer

TMB and MSI are considered predictive biomarkers of immune checkpoint inhibitor sensitivity^58,59^. To explore the potential role of YTHDF1 in pan-cancer, we first used the Wilcoxon test to compare YTHDF1 expression in pan-cancer. Then, the co-expression of YTHDF1 and pan-cancer was studied by considering TMB and MSI. Then, the stemness score of each sample was calculated according to the gene expression level for evaluating the relationship between m6A regulators and tumour stemness in pan-cancer. Finally, we applied the ESTIMATE and CIBERSORT methods to determine the pan-cancer role of YTHDF1 with respect to immune cell infiltration and the TME.

### Ethics approval and consent to participate

All patients were informed and written informed consent were provided. The study was conducted according to the clinical practice guidelines of the International Conference on Harmonization and the Declaration of Helsinki. This study protocol was approved by the ethical committee of Affiliated Hospital of Chongqing Medical University.

## Data Availability

The datasets used and/or analysed during the current study are available from the corresponding author on reasonable request.

## Abbreviations

BC: Breast cancer
m6A: N^6^-methyladenosine
TME: Tumour microenvironment
CNVs: Copy number of variants
CMap: Connectivity map
OS: Overall survival
GSVA: Gene set variation analysis
GSEA: Gene set enrichment analysis
qRT-PCR: Quantitative real-time reverse transcription polymerase chain reaction
TAM: Tumour-associated macrophages
